# Supplementary material for: The origin of the high electrochemical activity of pseudo-amorphous iridium oxides
Source: Nat Commun. 2021 Jun 24;12:3935. doi: 10.1038/s41467-021-24181-x (PMC8225786; doi:10.1038/s41467-021-24181-x)
Supplement: Supplementary file 1 — Supplementary information [file 41467_2021_24181_MOESM1_ESM.pdf]

Supplementary Information

The origin of the high electrochemical activity of pseudo-amorphous iridium oxides.

Marine Elmaalouf,[1@] Mateusz Odziomek,[2@] Silvia Duran,[3] Maxime Gayrard,[2] Mounib Bahri,[4] Cédric Tard,[3] Andrea Zitolo,[5] Benedikt Lassalle-Kaiser,[5] Jean-Yves Piquemal,[1] Ovidiu Ersen,[4] Cédric Boissière,[2] Clément Sanchez,[2] Marion Giraud,[1*] Marco Faustini[2*] and Jennifer Peron[1*]

[@]Equally contributed to the work

[1] Université de Paris, ITODYS, CNRS, UMR 7086, 15 rue J-A de Baïf, F-75013 Paris, France

[2] Sorbonne Université, CNRS, Collège de France, UMR 7574, Chimie de la Matière Condensée de Paris, F-75005 Paris, France.

[3] LCM, CNRS, Ecole Polytechnique, Institut Polytechnique de Paris, 91128 Palaiseau, France

[4] IPCMS-UMR 7504 CNRS, Université de Strasbourg, 23 rue du Loess, BP 43, 67034 Strasbourg Cedex 2, France

[5] Synchrotron SOLEIL, L'orme des Merisiers, BP 48 Saint-Aubin, 91192 Gif-sur-Yvette, France

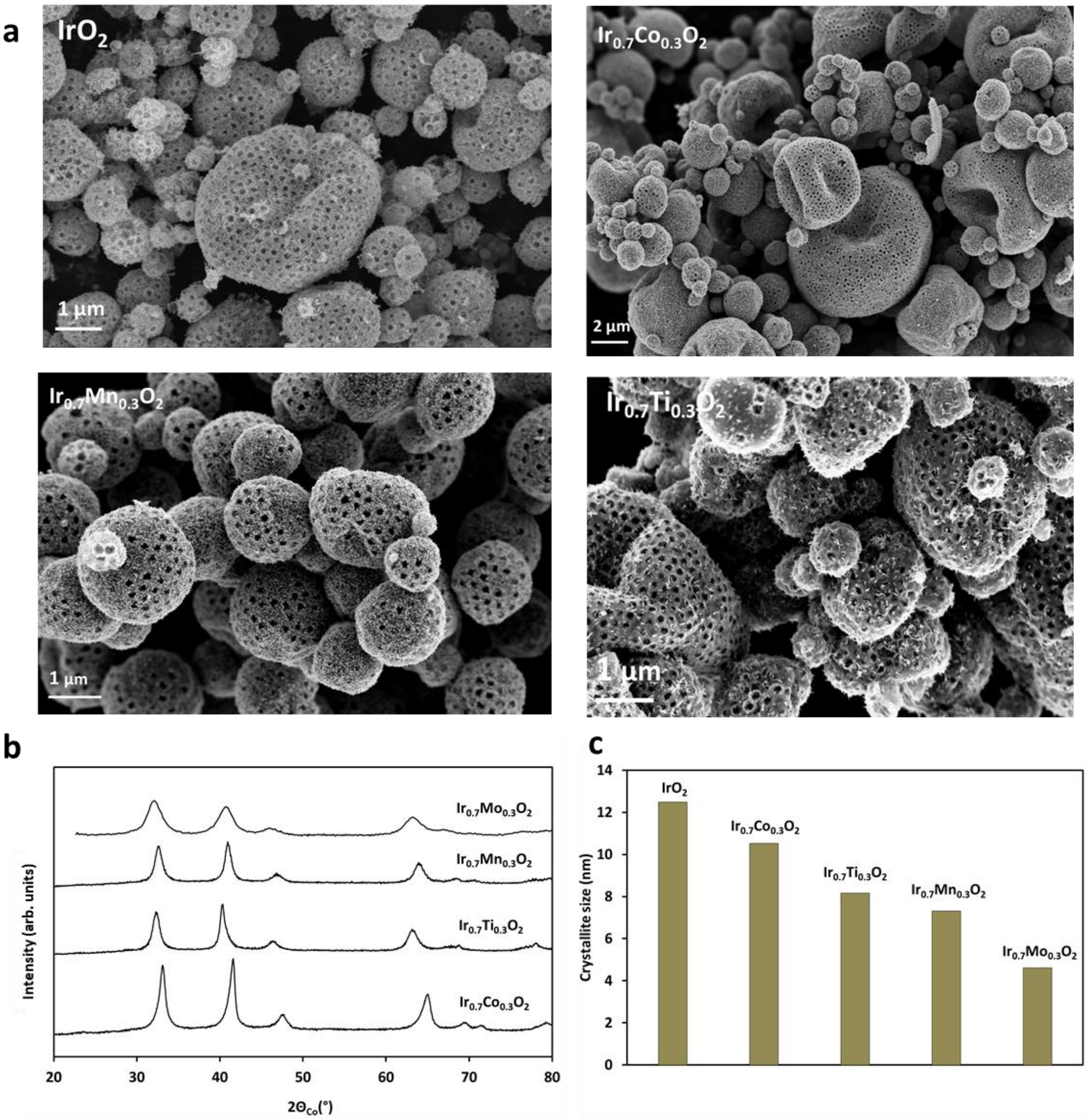

Supplementary Figure 1: (a) SEM images of the samples prepared with 100% Ir, and 70% Ir and 30% of Co, Mn or Ti and calcined at 550 °C; (b) X-ray diffractogramms of samples prepared with 70% of Ir and 30% of Co, Ti, Mn or Mo and calcined at 550 °C and (c) corresponding calculated crystallite size including IrO_2 from ref.[1]

All the materials prepared show a porous structure in which nanoparticles constitute the walls of macroporous microspheres. X-ray diffractograms show that at 550 °C with 30% of non-noble metal, regardless the non-noble metal used, the materials crystallize into one phase corresponding to a rutile-type structure. Calculated crystallite size shows that the particles obtained using Mo are much smaller than in the case of pure IrO_2 but also than that obtained with other metals. While all the materials already show peaks characteristics of rutile-type structure at 450 °C, peaks only appears after calcination at 550 °C for the Mo-containing material.

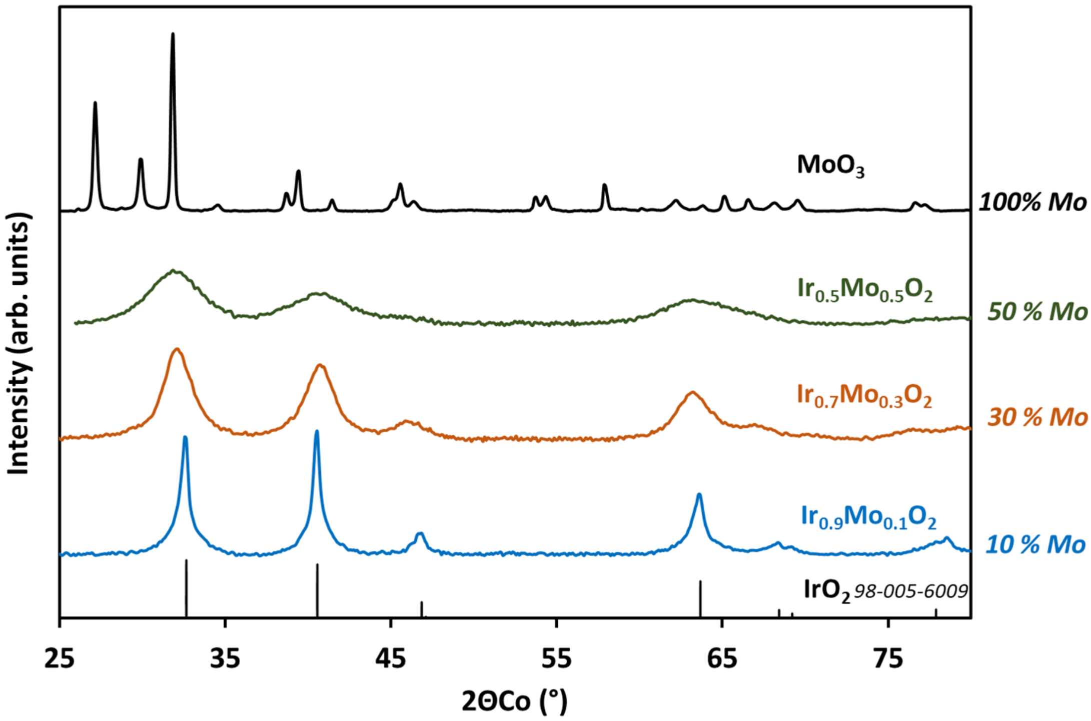

Supplementary Figure 2: X-ray diffractogramms of samples prepared with 10, 30 and 50% Mo and MoO_3, and calcined at 550 °C.

Samples prepared with 10 to 50% Mo crystallize into one single phase characteristic of rutile IrO_2-type structure (ICSD 98-008-4577). For a same calcination temperature of 550 °C, the peak width increases with Mo content, which indicates a decrease in crystallite size.

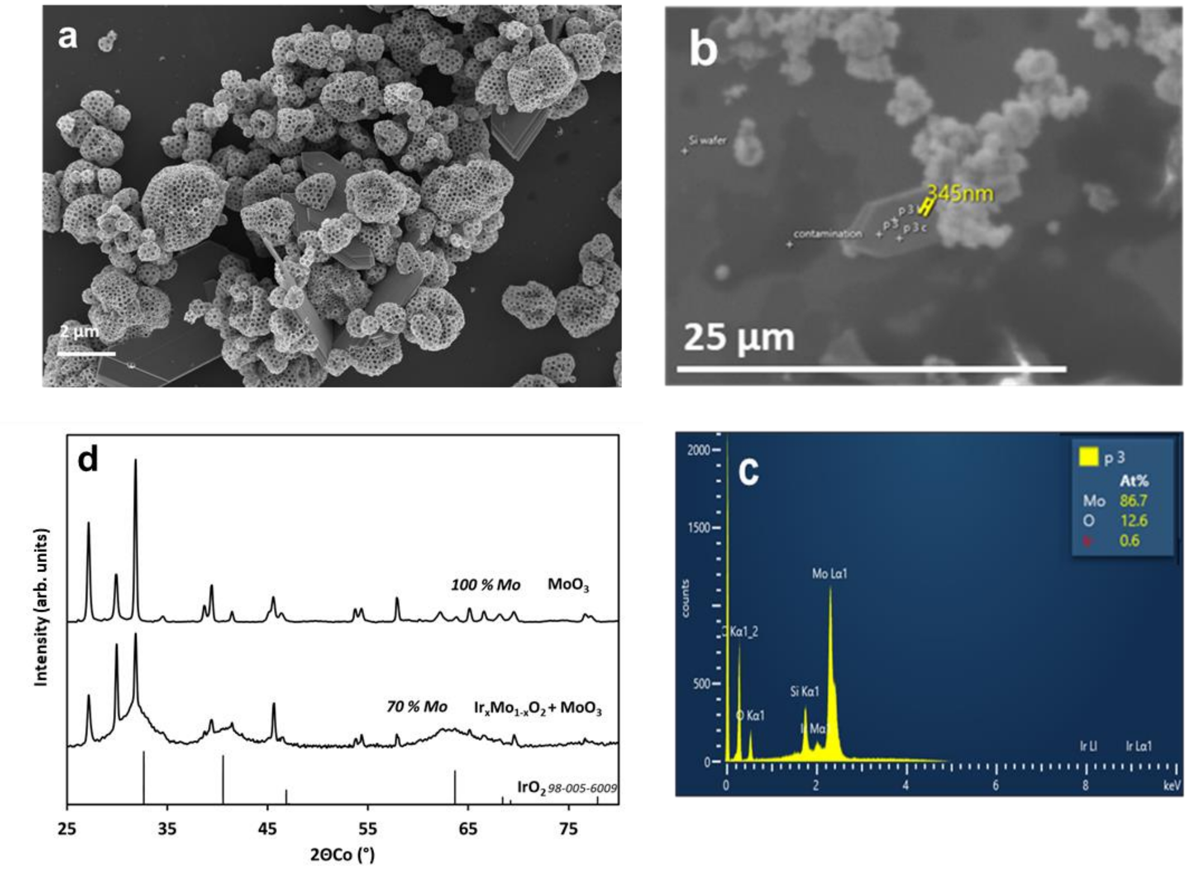

Supplementary Figure 3: (a) SEM image of a sample prepared with 70% Mo and calcined at 550 °C. (b, c) SEM image and corresponding EDX analysis of the highlighted platelet. (d) X-ray diffractogramms of the sample prepared with 70% Mo and of pure MoO_3, both calcined at 550 °C.

On SEM images of the sample prepared with 70% Mo and calcined at 550 °C, large platelets are observed along with macroporous microparticles. EDX analysis shows that the platelets only contain Mo and O (no Ir). Two phases are observed on the X-ray diffractogramms of the material calcined at 550 °C, one characteristic of IrO_2-rutile type structure (probably due to an IrMo mixed oxide forming microspheres) and one corresponding to MoO_3 (platelets).

Supplementary Table 1: Mo/(Mo+Ir) atomic percentage. Most active materials in each series is reported in bold character.

**Therotical Mo content**	**10%**		**30%**		**50%**	
**Calcination temperature (°C)**	% Mo XPS	% Mo XRF	% Mo XPS	% Mo XRF	% Mo XPS	% Mo XRF
**fresh**		10.3		30.4		50.7
**400**	12	9.4	46	29.6	52	49.6
**450**	**13**	**9.3**	34	30.4	62	48.8
**500**	16	7.5	**34**	**30.5**	48	50.1
**550**	15	7.7	35	30.5	**49**	**48.9**
**600**	12	7.4	35	30.2	50	49.3
**800**	17	9.8	31	31.2	38*	35.8*

For all the samples, the Mo content determined by Energy Dispersive X-Ray Fluorescence (EDXRF) correspond very well to the stoichiometry initially introduced in solution for the preparation of the materials. We observe a decrease in Mo content for the material initially containing 50% Mo upon calcination at 800 °C. This is probably due to a partial decomposition of the mixed oxide at T > 600 °C into $Ir_{0.35}Mo_{0.65}O_2$ (determined from the peak positions on the X-ray diffractogramm of the sample calcined at 800 °C) and MoO_3 (pure MoO_3 prepared by spray-drying was found to sublimate at T > 600 °C).

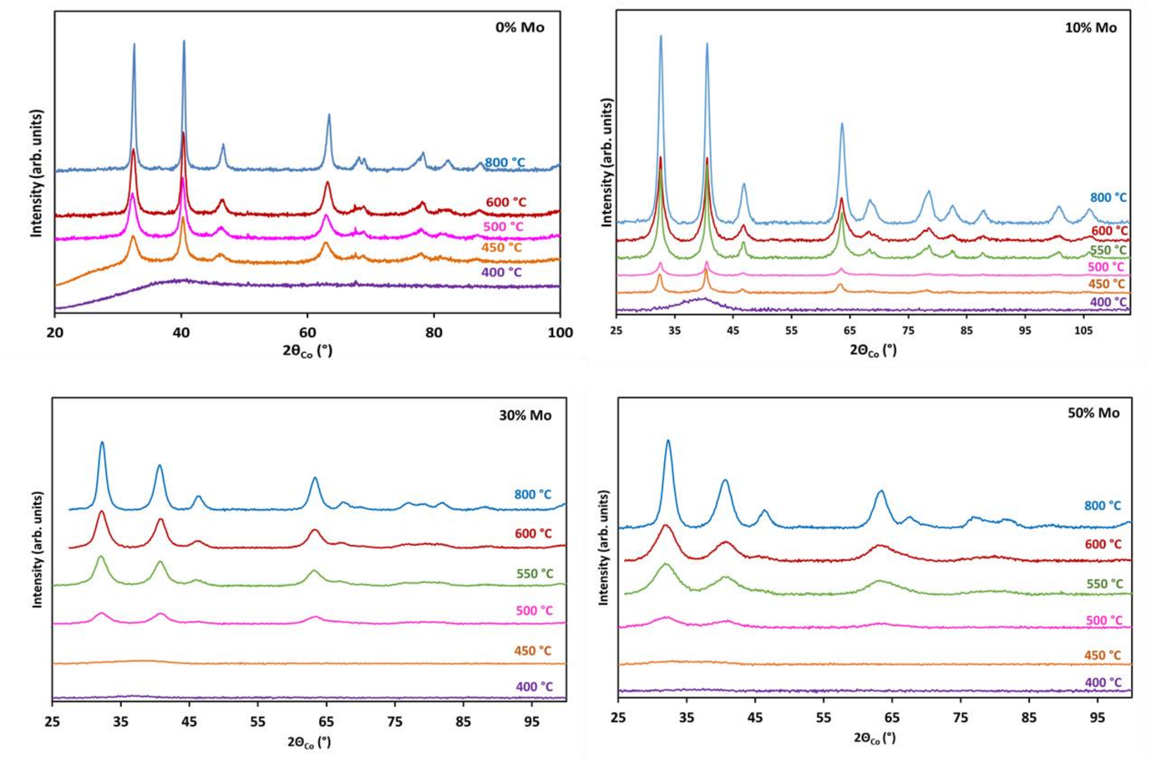

Supplementary Figure 4: X-ray diffractogramms of samples prepared with 0, 10, 30 and 50% Mo as a function of calcination temperature.

From these XRD, it can be seen that the higher the Mo content, the higher the crystallisation temperature and for a same calcination temperature, the smaller the crystallite size.

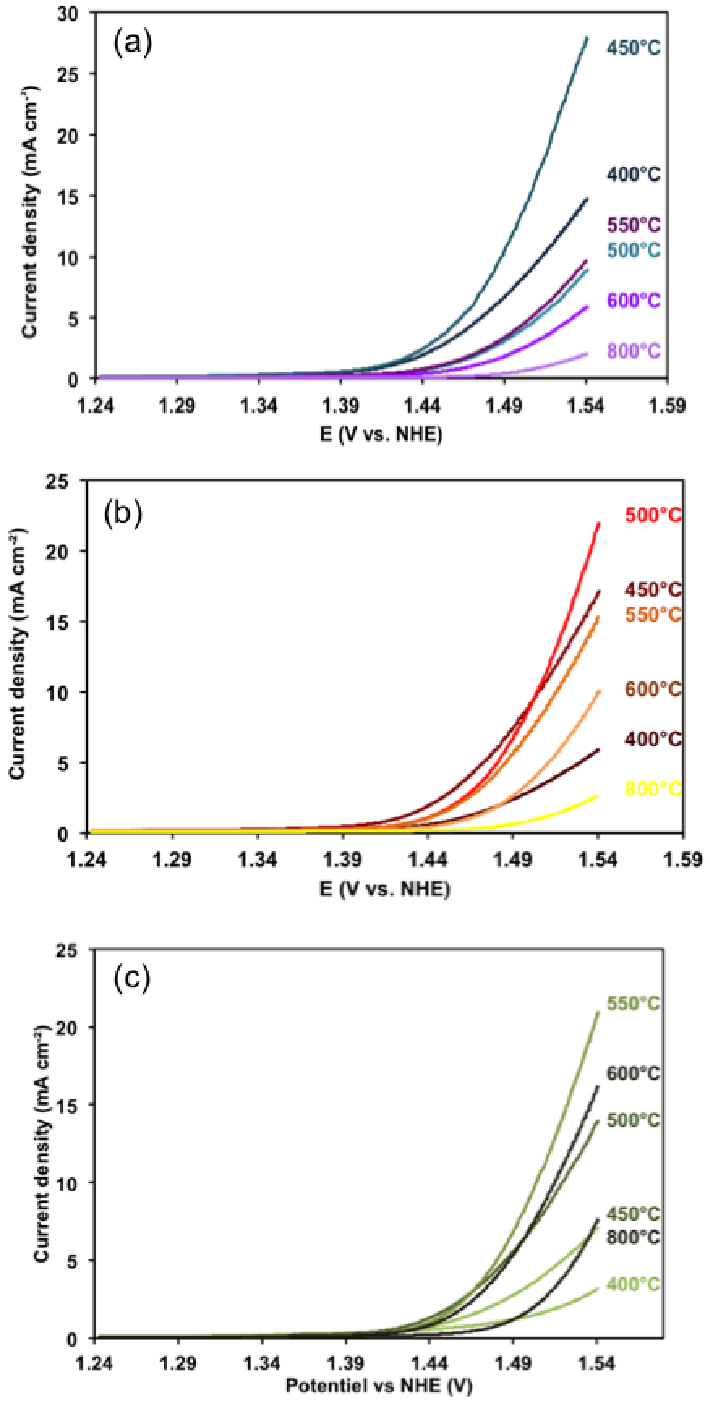

Supplementary Figure 5: Cyclic voltammograms of samples prepared with (a) 10% Mo, (b) 30% Mo and (c) 50% Mo and calcined from 400 to 800°C. Cyclic voltammograms (50th forward scan) were recorded at 10 mV s^{-1} in 0.05 M H_2SO_4; the results are normalized by the geometric surface of the electrode (total catalyst loading of the film: 0.0176 mg).

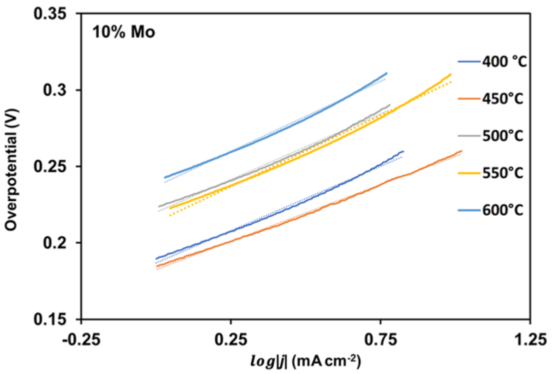

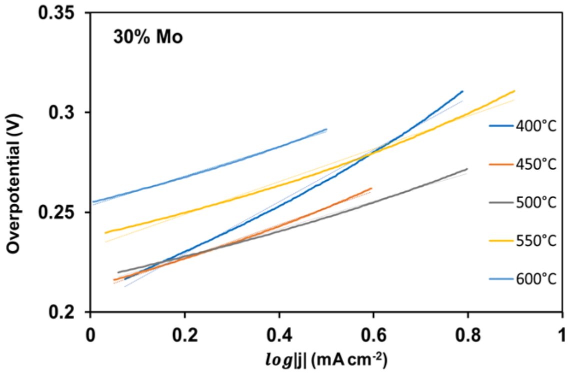

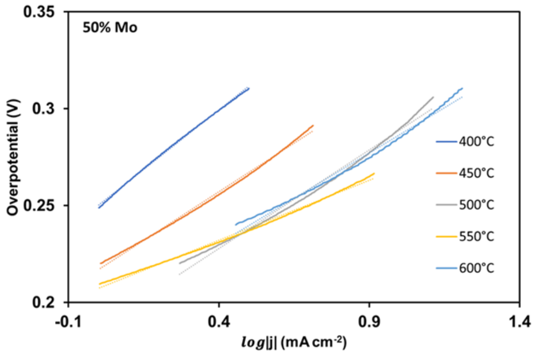

%Mo → / Temperature (°C) ↓	slope mV/dec 10% Mo	30% Mo	50% Mo
**400**	84.8	131.1	123.1
**450**	74.2	84.3	100.7
**500**	86.6	74.2	96.4
**550**	93.5	82.8	70.5
**600**	92.3	74.0	94.5

Supplementary Figure 6: Tafel plots represented for the 50th forward scan and calculated corresponding Tafel slopes of samples obtained after heat treatment in air at 400 °C, 450 °C, 500 °C, 550 °C and 600 °C. For most of samples, the Tafel slopes range from 70 and 90 mV/dec with a typical value of 70-74 for the most active materials of each series. The highest Tafel slopes are reported for highly amorphous materials.

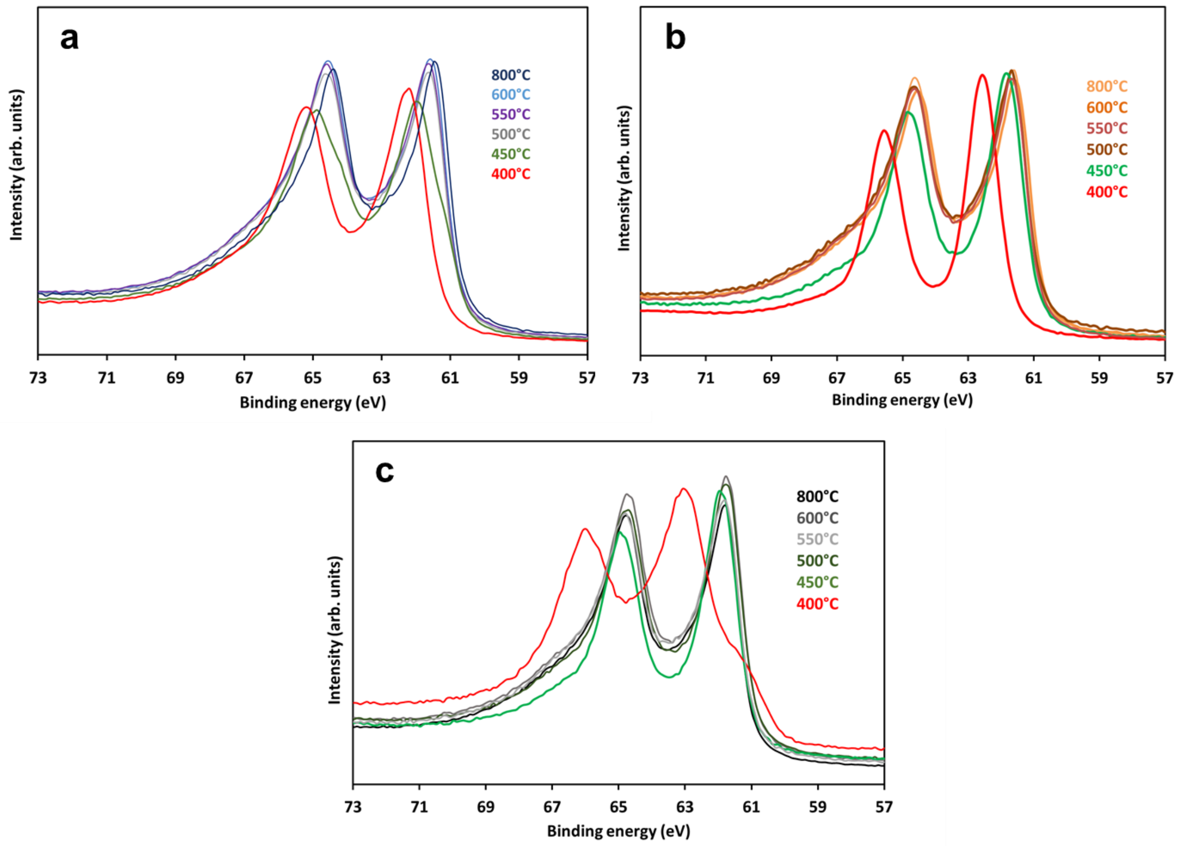

Supplementary Figure 7: XPS spectra in the Ir 4*f* region of samples prepared with (a) 10% Mo, (b) 30% Mo and (c) 50% Mo and calcined from 400 to 800°C.

Extensive studies of iridium oxidation states by XPS have been reported by Pfeifer et al. Coupling experimental with theoretical studies, these authors proposed that Ir(III) centres exhibit a reverse binding shift as already observed for Ag.[2-4] Ir(III) species usually show two peaks centered at 62.3 to 62.5 eV and 65.3 to 65.5 eV. As an electron conductor, IrO_2 has an asymmetric core level spectra, and asymmetric peaks characteristics of Ir(IV) in IrO_2 usually point at 61.7 to 61.9 eV and 64.7 to 64.9 eV for the $4f_{7/2}$ and $4f_{5/2}$ components, respectively. In our case, for the three samples, the XPS spectra of the powder calcined at 400 °C show two broad peaks centered at 62.3 and 65.3 eV that can be attributed to the presence of iridium (III) either resulting from residual chloro-iridium complexes or hydroxylated iridium species probably partially hydrated during the synthesis process. For samples calcined at 500 °C and above, all the spectra overlap and show two sharp peaks centered at 61.8 eV and 64.8 eV that can be attributed to the $4f_{7/2}$ and $4f_{5/2}$ components of the Ir(IV) in IrO_2 or $Ir_xMo_{1-x}O_2$. The oxidation of iridium species at the surface of the particles is already complete at 500 °C whatever the composition. For samples calcined at 450 °C, we observe an intermediate situation and samples obviously contain both Ir(III) and Ir(IV) species.

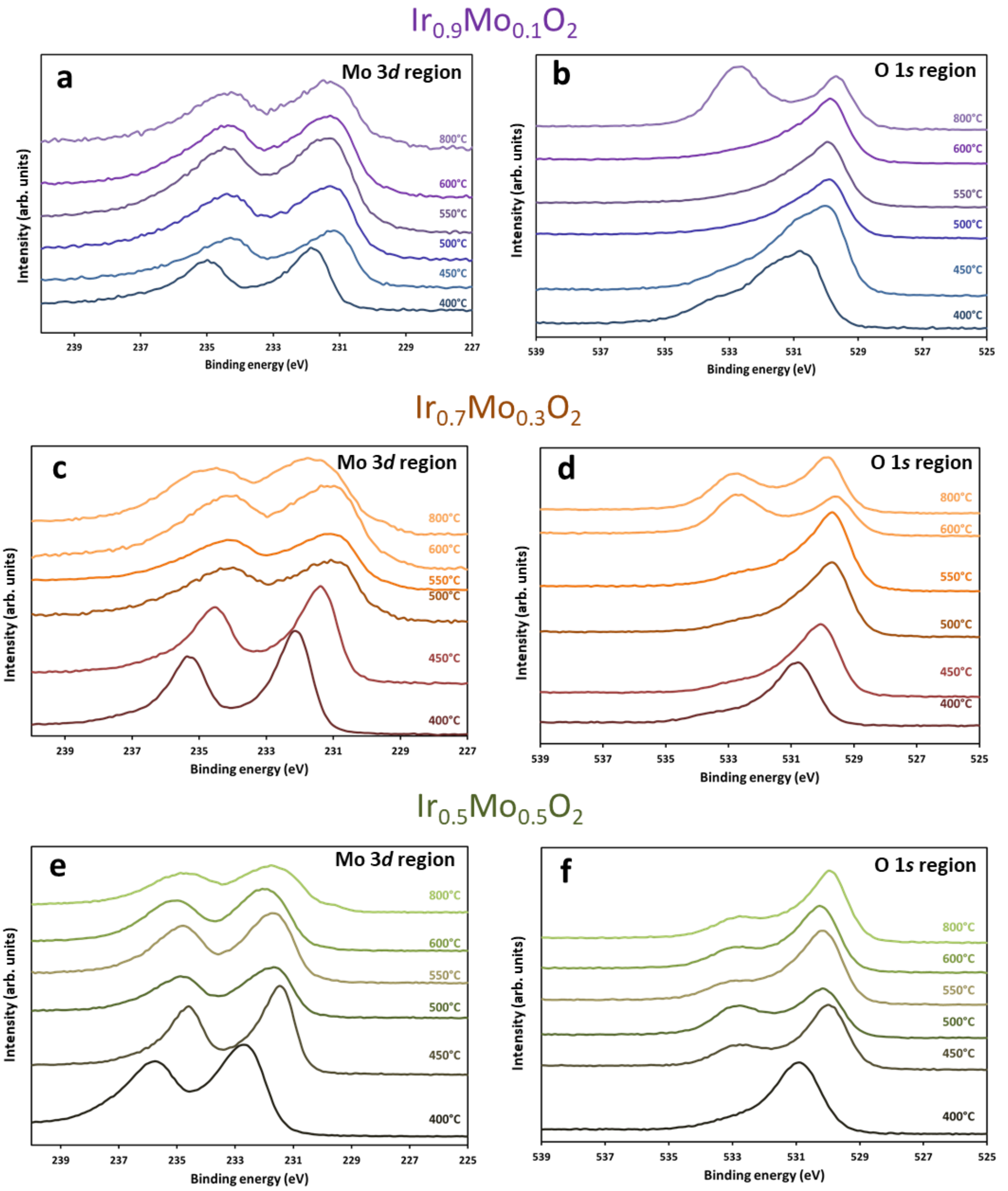

Supplementary Figure 8: XPS spectra in the Mo 3d region and O 1s region of samples prepared with (a,b) 10% Mo, (c,d) 30% Mo and (e,f) 50% Mo and calcined from 400 to 800°C.

In the Mo 3*d* region, after calcination at 400 °C, we observed two peaks located at 232.5 and 235.7 eV which are attributed to $3d_{5/2}$ and $3d_{3/2}$ spin-orbit components in Mo(VI), respectively. Upon heating, Mo(VI) is reduced into Mo(V) and $3d_{5/2}$ and $3d_{3/2}$ peaks are shifted to 231.5 and 234.7 eV, respectively. Mo remains in the same oxidation state up to 600 °C. At 800 °C, a small contribution of Mo(IV) characterized by a peak at 230 eV for the $3d_{5/2}$ component is observed along with Mo(V) peaks. In the O 1*s* spectra, at 400 °C, we mostly observe the peak centered at 531 eV characteristic of hydroxylated species. At 450 °C and above, the main contribution is centered at 530 eV and is characteristic of O-metal bonds; to a lesser extent a contribution of hydroxylated species is also observed. Another peak characteristic of water molecules is observed at 533 eV but is independent on the temperature of calcination and probably arises from residual water molecules adsorbed upon storage.

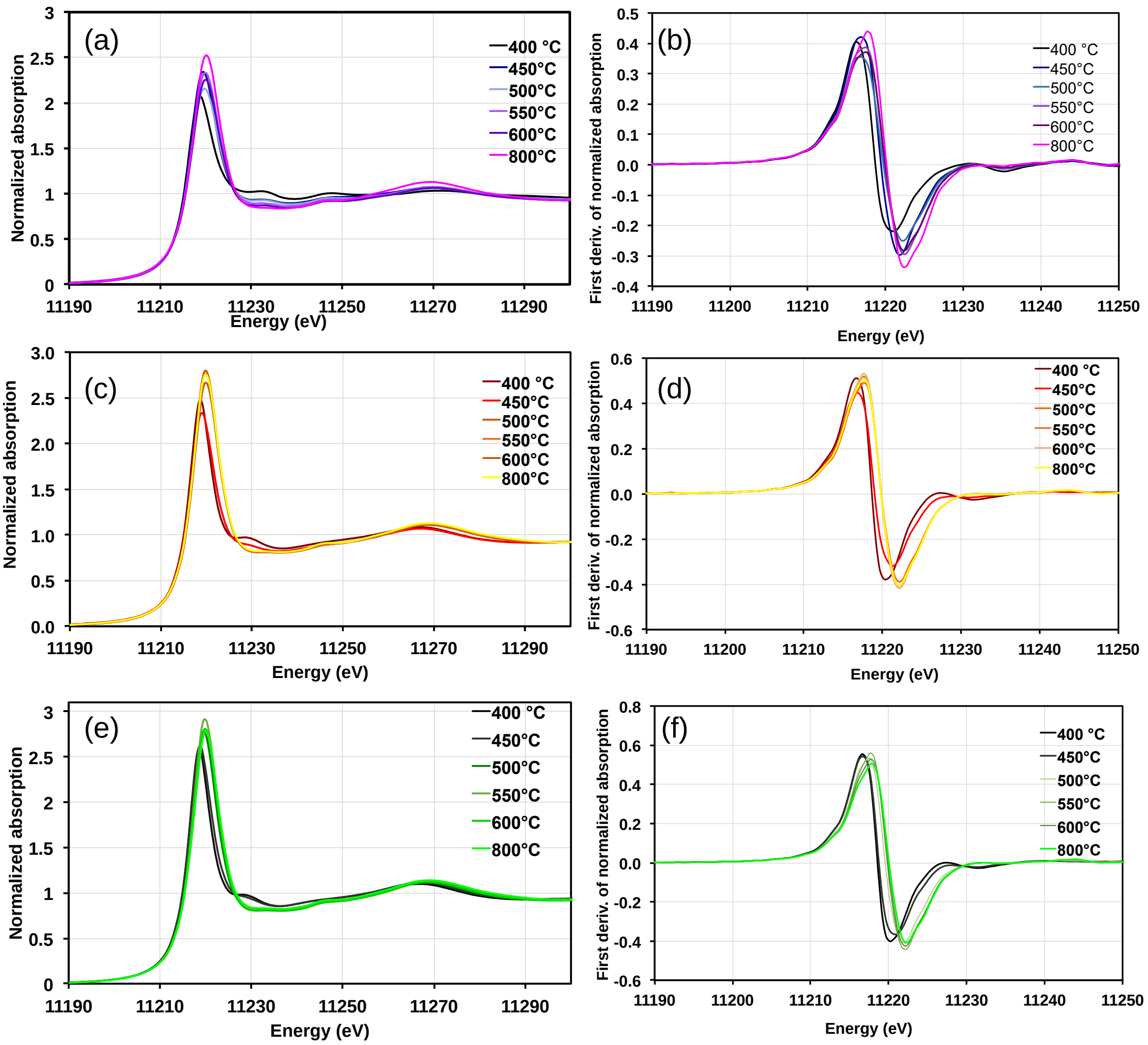

Supplementary Figure 9: XANES spectra at the Ir L_3 edge and the corresponding first derivative of samples prepared with (a,b) 10% Mo (c,d) 30% Mo and (e,f) 50% Mo and calcined from 400 to 800 °C.

Whatever the Mo content, the shift of the peak of the first derivative occurs between 450 and 500 °C.

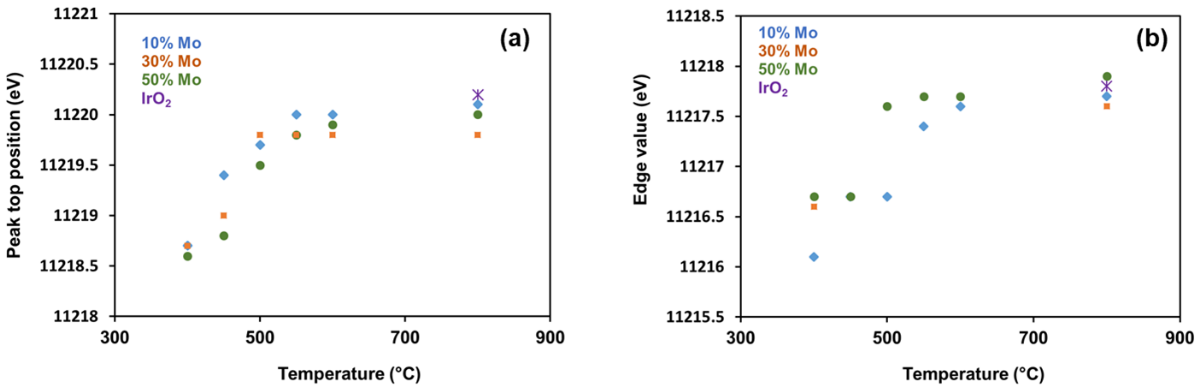

Supplementary Figure 10: (a) Peak-top and (b) edge values determined from XANES spectra at the Ir L_3 edge with 10% Mo, 30% Mo and 50% Mo and calcined from 400 to 800 °C.

To probe an electronic influence of Mo onto Ir, we have plotted the position of the edge and the peak top position (commonly referred as white line) determined on iridium L_3 edge XANES spectra of each compound calcined at each temperature. On these two plots, we do not observe a particular trend of the top peak position or of edge value when Mo content in the material increases. Whatever the composition, for calcination temperature higher than 500 °C *i.e.* when there is no influence of the oxidation process, the edge and white positions ranges from 11217.5 eV and 11218 eV and 11219.8 and 11220.2 eV, respectively. Even if it is more difficult to draw affirmative conclusions from the samples calcined between 400 and 500 °C due to the concomitant oxidation, similar observation is made in this temperature range and the relative position of the points do not seem to depend on Mo content. This rules out a strong influence of Mo on Ir electronic structure. However, we clearly see that the edge and peak top positions increase of *ca.* 1 eV when the calcination temperature increases from 400 to 500 °C indicating the transformation of Ir(III) into Ir(IV) species upon heating.

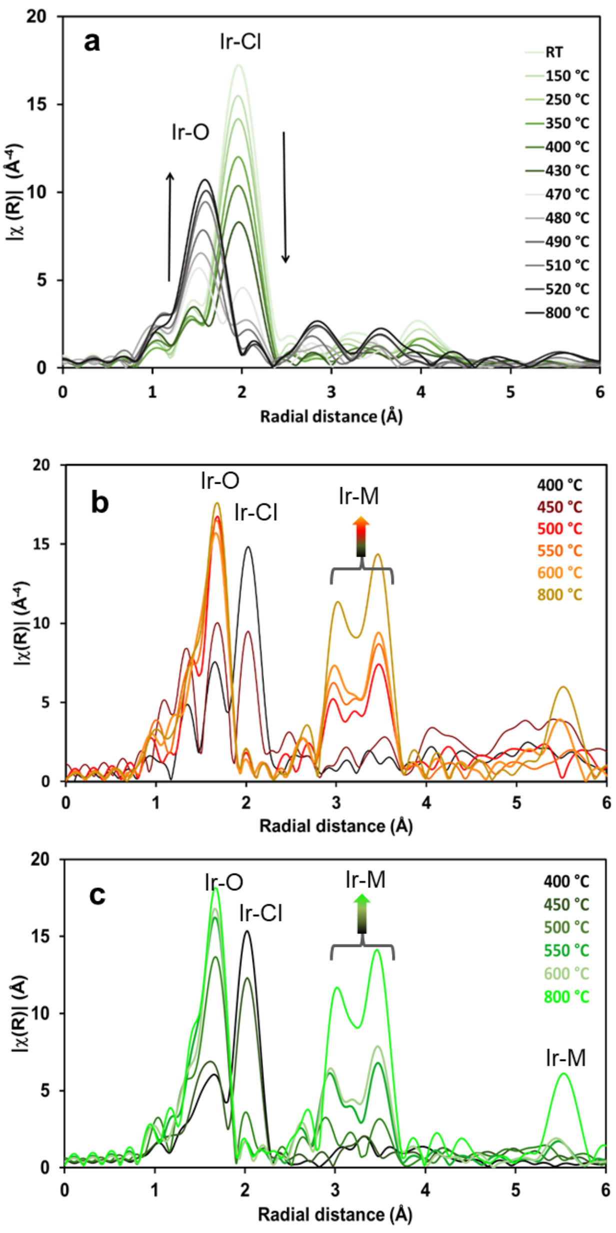

Supplementary Figure 11: (a) Temperature-resolved Fourier transform of the Ir L_3-edge EXAFS spectra for pure Ir-sample upon calcination. Fourier transform of the Ir L_3-edge EXAFS spectra for the sample prepared with (b) 30% Mo and (c) 50% and calcined at various temperatures.

Between 400 and 450 °C, the Ir-Cl peak at R' = 1.99 Å (uncorrected for phase shift) disappears and the intensity of the peak characteristic of Ir-O at R' = 1.65 Å increases. As the temperature increases, the peaks characteristic of Ir-Ir interaction appear at R' = 2.91 Å and 3.55 Å and grow as the calcination temperature increases due to the crystallization of the material. As the Mo content increases, the temperature at which the peak characteristic of Ir-Ir interaction appears increases. For the sample prepared with 50% of Mo, below 550 °C (optimal calcination temperature), only Ir-O bonds are visible characteristic of an amorphous structure; above this optimal temperature, two peaks characteristic of Ir-M (M= Ir, Mo) interactions (observed at 2.97 and 3.60 Å) increase in intensity which indicates a longer-range ordering. The presence of the additional peak at 3.11 Å, not observed for pure IrO_2, strongly supports the other analyses on the presence of Mo in the rutile lattice of Ir-based oxide.

Supporting Table 2:

Supporting Table 2a: Summary of the EXAFS fit Parameters for the pure IrO_2 calcined at different temperatures and recorded at the calcination temperature (except the 800 °C sample which was also recorded at RT.)[a]

Sample	Shell	N	R / Å	σ^2 / Å^2	ΔE0 / eV	R_f (%)	χ^2red
0% 400 °C	Ir-O	1.10 *0.38*	2.00 *0.05*	**0.006 (fixed)**	7.98 *1.54*	0.77	767.95
	Ir-Cl	4.44 *0.33*	2.35 *0.01*	**0.005 (fixed)**			
0% 450 °C	Ir-O	2.52 *0.18*	1.97 *0.01*	**0.006 (fixed)**	7.52 *0.80*	0.41	387.99
	Ir-Cl	3.08 *0.16*	2.34 *0.01*	**0.006 (fixed)**			
0% 500 °C	Ir-O	5.74 *0.44*	1.98 *0.01*	0.006 *0.001*	10.30 *0.89*	0.75	363.68
0% 550 °C	Ir-O	5.84 *0.45*	1.98 *0.01*	0.004 *0.001*	10.85 *0.94*	1.20	571.49
0% 650 °C	Ir-O	5.79 *0.80*	1.98 *0.01*	0.004 *0.001*	10.86 *1.61*	3.87	947.25
0% 800 °C	Ir-O	**6 (fixed)**	1.97 *0.01*	0.005 *0.001*	10.26 *0.82*	0.67	137.54
0% 800 °C (at RT)[b]	Ir-O	**6 (fixed)**	1.98 *0.01* *(DRX 2.00)*	0.002 *0.001*	10.87 *0.75*	1.52	4442.11
	Ir-Ir_1	**2 (fixed)**	3.14 *0.01* *(DRX 3.16)*	0.002 *0.001*			
	Ir-O_2	**4 (fixed)**	3.56 *0.03* *(DRX 3.41)*	0.004 *0.004*			
	Ir-Ir_2	**8 (fixed)**	3.54 *0.01* *(DRX 3.56*	0.001 *0.001*			

[a] N, coordination numbers, r, distances between Ir and neighboring atoms, σ, Debye Waller factors, ΔE_0, scaling energy parameter and R_f goodness of fit. Bold numbers correspond to fixed values and uncertainties are given in italic. Those parameters were obtained by least square fitting over a 1.0-3.0 Å range of the Fourier transforms of the experimental EXAFS spectra (k_{min} = 2.8 Å^{-1} and k_{max} = 10.6 Å^{-1}, Hanning window with dk = 1). The reliability of the fit was assessed by the minimization of the reduced χ^2_{red} and metrics defined by the IXS standards and criteria committee (http://ixs.iit.edu/subcommittee_reports/sc/err-rep.pdf).

[b] The fit of this sample was used to determine the value of the amplitude reduction factor S_0^2 = 0.80. The mean distances determined from the crystallographic structure for IrO_2 are reported for comparison purpose. Those parameters were obtained by least square fitting of over 1.1-4.0 Å of the Fourier transforms of the experimental EXAFS spectra (k_{min} = 3.0 Å^{-1} and k_{max} = 14.0 Å^{-1}, Hanning window with dk = 1).

Supporting Table 2b: Summary of the EXAFS fit Parameters for the 30% Mo series calcined at different temperatures and recorded at RT.[a]

Sample	Shell	N	R / Å	σ^2 / Å²	ΔE_0 / eV	R_f (%)	χ^2red
30% 400 °C	Ir-O	2.11 *0.53*	2.02 *0.02*	0.003 *0.002*	10.13 *0.83*	1.76	1129.68
	Ir-Cl	3.80 *0.49*	2.35 *0.01*	0.004 *0.001*			
30% 450 °C	Ir-O	2.85 *0.86*	2.01 *0.02*	0.002 *0.002*	10.27 *1.82*	12.34	1179.96
	Ir-Cl	2.05 *1.02*	2.35 *0.02*	0.004 *0.004*			
30% 500 °C	Ir-O	5.56 *0.37*	1.99 *0.01*	0.002 *0.001*	10.44 *0.87*	4.21	1127.15
30% 550 °C	Ir-O	5.79 *0.29*	1.98 *0.01*	0.003 *0.001*	10.26 *0.67*	1.85	894.59
30% 600 °C	Ir-O	5.98 *0.30*	1.98 *0.01*	0.003 *0.001*	10.03 *0.65*	1.60	2079.70
30% 800 °C	Ir-O	5.89 *0.29*	1.98 *0.01*	0.002 *0.001*	10.64 *0.64*	1.54	2232.25

[a] N, coordination numbers, r, distances between Ir and neighboring atoms, σ, Debye Waller factors, ΔE_0, scaling energy parameter and R_f goodness of fit. Bold numbers correspond to fixed values and uncertainties are given in italic. Those parameters were obtained by least square fitting over a 1.0-3.0 Å range of the Fourier transforms of the experimental EXAFS spectra (k_{min} = 2.8 Å^{-1} and k_{max} = 10.6 Å^{-1}, Hanning window with dk = 1).

Supporting Table 2c: Summary of the EXAFS fit Parameters for the 50% Mo series calcined at different temperatures and recorded at RT.[a]

Sample	Shell	N	R / Å	σ^2 / Å²	ΔE0 / eV	R_f (%)	χ^2red
50% 400 °C	Ir-O	2.18 *0.25*	2.01 *0.01*	0.003 *0.001*	10.48 *0.41*	2.73	88.88
	Ir-Cl	3.73 *0.21*	2.35 *0.01*	0.004 *0.001*			
50% 450 °C	Ir-O	3.59 *0.25*	2.00 *0.01*	0.005 *0.001*	10.47 *0.35*	2.69	159.42
	Ir-Cl	2.66 *0.18*	2.35 *0.01*	0.003 *0.001*			
50% 500 °C (Oxygens only)	Ir-O	5.76 *0.24*	1.99 *0.01*	0.003 *0.001*	10.87 *0.53*	1.35	851.11
50% 500°C	Ir-O	5.60 *0.32*	1.99 *0.01*	0.003 *0.001*	10.79 *0.59*	1.12	993.43
	Ir-Cl	0.15 *0.21*	2.34 *0.05*	0.001 *0.001*			
50% 550 °C	Ir-O	6.03 *0.28*	1.98 *0.01*	0.003 *0.001*	10.53 *0.60*	1.35	1453.51
50% 600 °C	Ir-O	5.76 *0.31*	1.99 *0.01*	0.002 *0.001*	10.94 *0.70*	1.52	1027.26
50% 800 °C	Ir-O	5.90 *0.27*	1.98 *0.01*	0.002 *0.001*	10.74 *0.60*	1.19	281.97

[a] N, coordination numbers, r, distances between Ir and neighboring atoms, σ, Debye Waller factors, ΔE_0, scaling energy parameter and R_f goodness of fit. Bold numbers correspond to fixed values and uncertainties are given in italic. Those parameters were obtained by least square fitting over a 1.0-3.0 Å range of the Fourier transforms of the experimental EXAFS spectra (k_{min} = 2.8 Å^{-1} and k_{max} = 10.6 Å^{-1}, Hanning window with dk = 1).

Supporting Table 3: Cl/(Mo+Ir) atomic ratio determined from XPS analysis. Values calculated for the most active materials in each series are reported in bold character.

calcination temperature (°C)	Cl/(Mo+Ir)			
	10% Mo	30% Mo	50% Mo	pure Ir
400	0.32	0.56	0.17	0.38
450	**0.14**	0.17	0.12	**0.21**
500	0.05	**0.03**	0.04	0.20
550	0.03	0.02	**0.06**	0.13
600	0.03	0.02	0.04	0.15
800	0.05	0.02	0.03	

Supporting Table 4: Crystallite size determined by Retvield refinement of the corresponding X-ray diffractogramms as a function of calcination temperature. Most active materials in each series is reported in bold character. *The crystallite size determined for pure IrO_x materials is an average between size calculated from *a* and from *c* parameters (see ref.[1] for details).

Calcination temperature (°C)	Crystallite size (nm)			
	10% Mo	30% Mo	50% Mo	0% Mo
400	n.a.	n.a.	n.a.	n.a.
450	**1.5**	n.a.	n.a.	**8.6***
500	6	**2.5**	n.a.	10*
550	7	4	**2**	12.5*
600	7.5	4.5	2.5	15.8*
800	11	7	5	27.7*

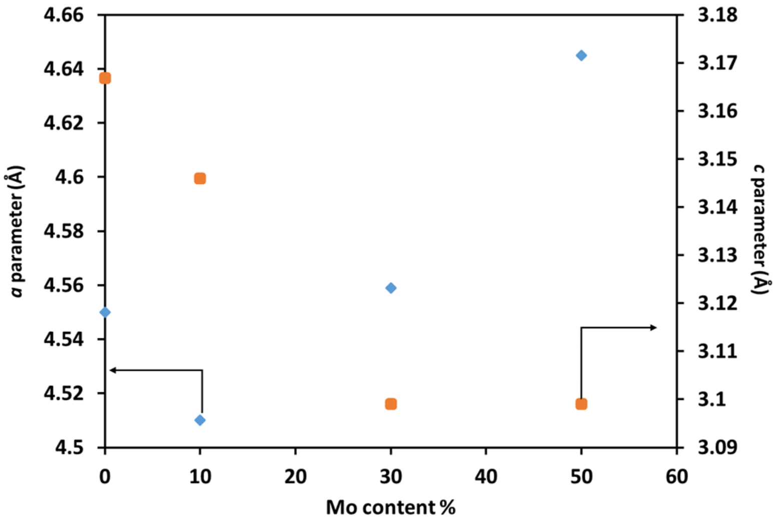

Supplementary Figure 12: Evolution of *a* (blue diamonds) and *c* (orange squares) unit-cell parameters as a function of Mo content for samples calcined at 600 °C.

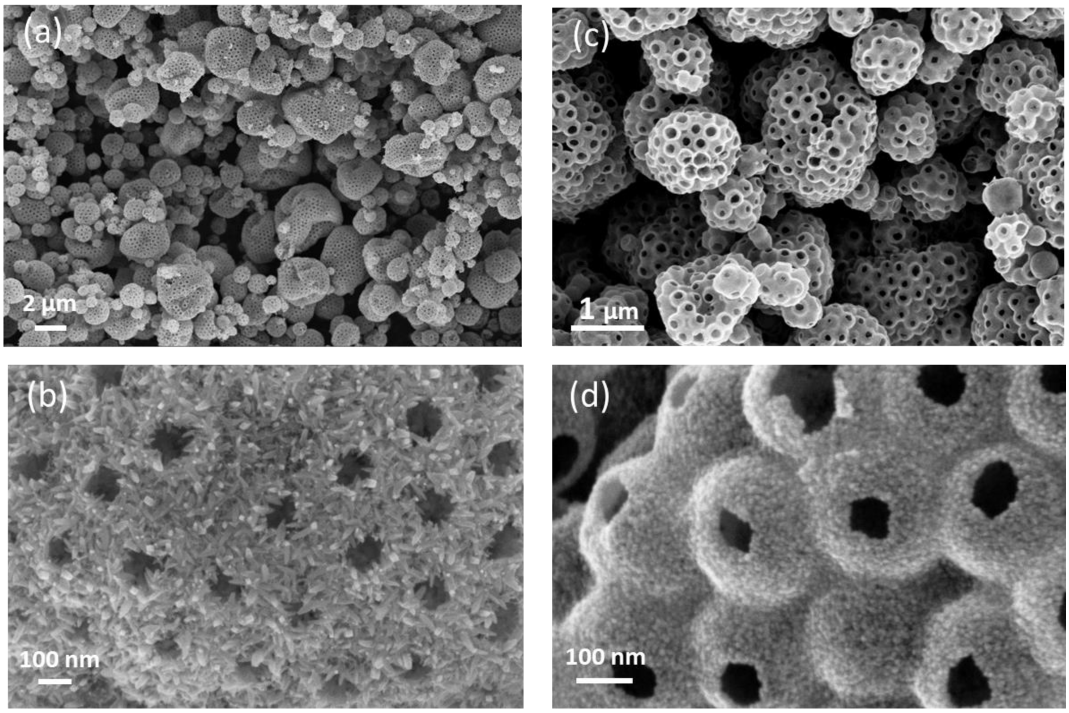

Supplementary Figure 13: SEM micrographs of IrO_2 (a,b) and $Ir_{0.7}Mo_{0.3}O_2$ (c,d) samples calcined at 500 °C. These micrographs illustrate the differences between the particles shape of pure IrO_2 which crystallizes into strongly anisotropic nanoneedles, and the mixed oxide forming isotropic nanoparticles.

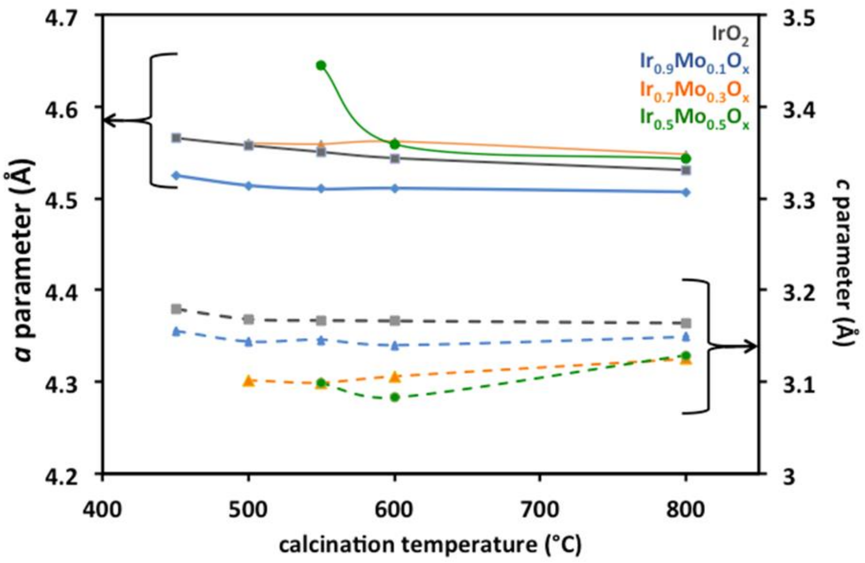

Supplementary Figure 14: Evolution of *a* and *c* parameters as a function of the calcination temperature.

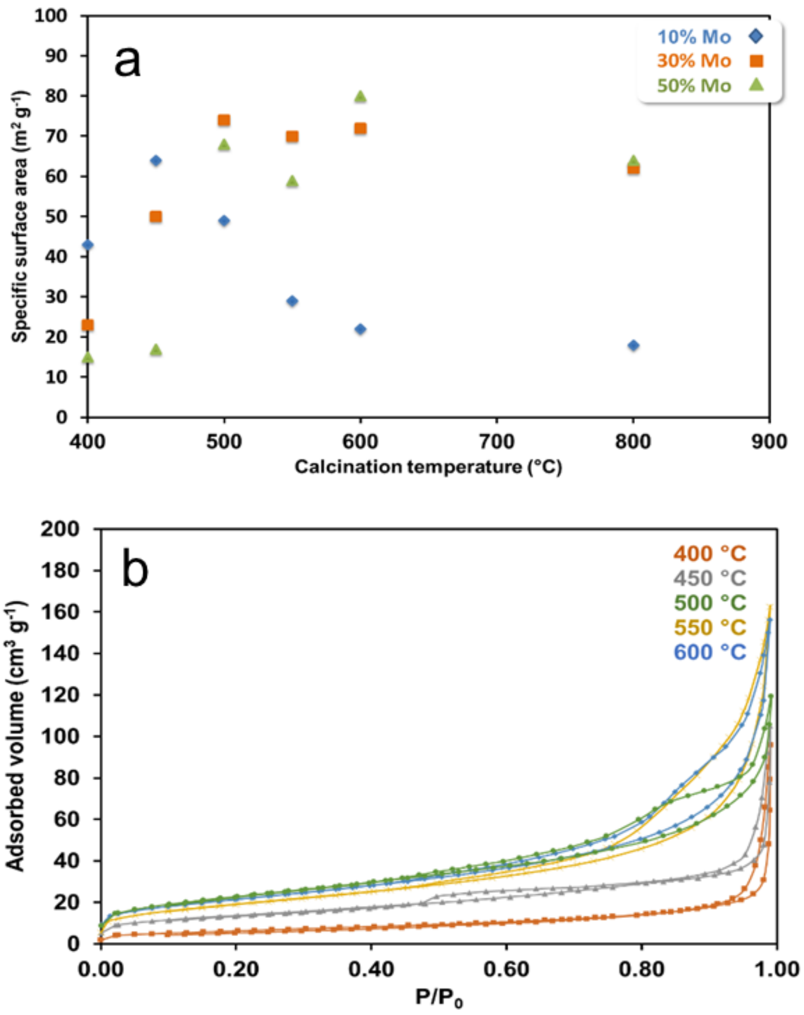

Supplementary Figure 15: (a) Specific surface area determined using BET equation of the samples prepared with various Mo content and calcined from 400 °C to 800 °C, (b) Isotherms obtained for the series containing 30% Mo and calcined at different temperature.

1 Faustini, M. *et al.* Hierarchically Structured Ultraporous Iridium-Based Materials: A Novel Catalyst Architecture for Proton Exchange Membrane Water Electrolyzers. *Advanced Energy Materials* 9, 1802136, doi:10.1002/aenm.201802136 (2019).

2 Pfeifer, V. *et al.* The electronic structure of iridium and its oxides. *Surface and Interface Analysis* 48, 261–273, doi:10.1002/sia.5895 (2015).

3 Pfeifer, V. *et al.* The electronic structure of iridium oxide electrodes active in water splitting. *Physical Chemistry Chemical Physics* 18, 2292-2296, doi:10.1039/C5CP06997A (2016).

4 Pfeifer, V. *et al.* Reactive oxygen species in iridium-based OER catalysts. *Chemical Science* 7, 6791-6795, doi:10.1039/C6SC01860B (2016).
